# Association of lower level of leisure-related physical activity with primary headaches

**DOI:** 10.1186/1129-2377-1-S14-P205

**Published:** 2013-02-21

**Authors:** S Ashina, L Bendtsen, AC Lyngberg, RB Lipton, N Hajiyeva, RH Jensen

**Affiliations:** 1 Pain Medicine, Palliative Care, Neurology, Albert Einstein College of Medicine, Beth Israel Medical Center, USA; 2 Danish Headache Center, Neurology, University of Copenhagen, Glostrup Hospital, Denmark; 3 National Board of Health, Copenhagen, Denmark; 4 Neurology, Albert Einstein College of Medicine, Montefiore Headache Center, USA; 5 Pain Medicine and Palliative Care, Albert Einstein College of Medicine, Beth Israel Medical Center, USA

## Background and aims

Low physical activity has been associated with higher prevalence of headaches. The primary aim of the study was to assess the association of pure migraine, pure tension-type headache (TTH) and coexistent headache with the level of leisure-related physical activity. The secondary aim was to study the association between the level of leisure-related physical activity and episodic and chronic mixed headache.

## Methods

Total of 1300 subjects was invited to participate in a cross-sectional population study. A total of 805 eligible subjects completed a diagnostic headache interview, provided self-reported data on neck pain and back pain, leisure-related physical activity, demographics and self-rated health. Levels of leisure-related physical activity were classified according to activities performed as low, medium and high (reference).

## Results

Multinomial logistic regression analysis adjusted for gender, age, education, neck pain, back pain, poor self-rated health demonstrated that primary headache (mixed headache ) was associated low physical activity (OR=2.54, 95% CI=1.47-4.39, p=0.001) followed by medium physical activity (OR=1.62, 95% CI=1.05-2.52, p=0.03). Low physical activity was significantly associated with pure TTH (OR=2.80, 95%CI=1.38-5.68, p=0.004) and coexistent headache (OR=2.97, 95%CI=1.27-6.99, p=0.01) in the adjusted analysis. Association of pure migraine with low (OR=1.95, 95% CI=0.86-4.43, p=0.11) and medium physical activity, and coexistent headache and pure TTH with medium physical activity did not reach statistical significance. Low activity level is highest in chronic headache (OR=2.97, 95%CI=0.94-9.44, p=0.07) compared to episodic headache (OR=2.42, 95%CI=1.37-4.28, p=0.002).

## Conclusions

Lower level of leisure-related physical activity is associated with primary headaches. Furthermore, the association is strongest for coexistent headache followed by pure TTH and pure migraine. The causal relationship of low activity level to headache chronicity cannot be assessed in this cross-sectional study.

